# Glucose metabolite methylglyoxal induces vascular endothelial cell pyroptosis via NLRP3 inflammasome activation and oxidative stress in vitro and in vivo

**DOI:** 10.1007/s00018-024-05432-8

**Published:** 2024-09-13

**Authors:** Yanan Wang, Jinxiang Chen, Youkun Zheng, Jun Jiang, Liqun Wang, Jianbo Wu, Chunxiang Zhang, Mao Luo

**Affiliations:** 1 Basic Medicine Research Innovation Center for Cardiometabolic DiseasesMinistry of EducationLaboratory for Cardiovascular Pharmacology, Department of Pharmacology, School of Pharmacy, Southwest Medical University, Luzhou, Sichuan China; 2 Municipal Key Laboratory of Thrombosis and Vascular Biology, Luzhou, Sichuan China; 3 Clinical Research Center (CRC), Clinical Pathology Center (CPC), Cancer Early Detection and Treatment Center (CEDTC) and Translational Medicine Research Center (TMRC), Chongqing University Three Gorges Hospital, Chongqing University, Wanzhou, Chongqing, China; 4 Department of General Surgery (Thyroid Surgery), the Affiliated Hospital of Southwest Medical University, Luzhou, Sichuan China; 5 Metabolic Vascular Diseases Key Laboratory of Sichuan Province, Luzhou, Sichuan China

**Keywords:** Sulforaphane, MGO, ROS, NLRP3 inflammasome, Pyroptosis

## Abstract

Methylglyoxal (MGO), a reactive dicarbonyl metabolite of glucose, plays a prominent role in the pathogenesis of diabetes and vascular complications. Our previous studies have shown that MGO is associated with increased oxidative stress, inflammatory responses and apoptotic cell death in endothelial cells (ECs). Pyroptosis is a novel form of inflammatory caspase-1-dependent programmed cell death that is closely associated with the activation of the NOD-like receptor 3 (NLRP3) inflammasome. Recent studies have shown that sulforaphane (SFN) can inhibit pyroptosis, but the effects and underlying mechanisms by which SFN affects MGO-induced pyroptosis in endothelial cells have not been determined. Here, we found that SFN prevented MGO-induced pyroptosis by suppressing oxidative stress and inflammation in vitro and in vivo. Our results revealed that SFN dose-dependently prevented MGO-induced HUVEC pyroptosis, inhibited pyroptosis-associated biochemical changes, and attenuated MGO-induced morphological alterations in mitochondria. SFN pretreatment significantly suppressed MGO-induced ROS production and the inflammatory response by inhibiting the NLRP3 inflammasome (NLRP3, ASC, and caspase-1) signaling pathway by activating Nrf2/HO-1 signaling. Similar results were obtained in vivo, and we demonstrated that SFN prevented MGO-induced oxidative damage, inflammation and pyroptosis by reversing the MGO-induced downregulation of the NLRP3 signaling pathway through the upregulation of Nrf2. Additionally, an Nrf2 inhibitor (ML385) noticeably attenuated the protective effects of SFN on MGO-induced pyroptosis and ROS generation by inhibiting the Nrf2/HO-1 signaling pathway, and a ROS scavenger (NAC) and a permeability transition pore inhibitor (CsA) completely reversed these effects. Moreover, NLRP3 inhibitor (MCC950) and caspase-1 inhibitor (VX765) further reduced pyroptosis in endothelial cells that were pretreated with SFN. Collectively, these findings broaden our understanding of the mechanism by which SFN inhibits pyroptosis induced by MGO and suggests important implications for the potential use of SFN in the treatment of vascular diseases.

**Supplementary Information:**

The online version contains supplementary material available at 10.1007/s00018-024-05432-8.

## Introduction

Vascular disease is one of the most prevalent causes of morbidity and mortality associated with diabetes mellitus (DM) [1–3]. Vascular endothelial cells (ECs), which form the inner lining of all blood vessels, can protect transport logistics, regulate vascular tone, control vascular permeability and provide a crucial link between the cardiovascular system and the immune system [4–6]. However, intravascular homeostasis disorders caused by EC injury and dysfunction are the major causes of vascular complications in DM [7–9]. Several studies have suggested that ECs are subjected to oxidative stress and inflammatory reactions and that these processes can perturb cellular functions involved in intracellular signal transduction pathways, resulting in pyroptosis [8, 10–12].

Methylglyoxal (MGO) is a highly reactive electrophilic α, β-dicarbonyl aldehyde compound produced by glycolysis and is the main precursor of advanced glycation end-product (AGE) [13–15]. Our previous studies and others have demonstrated that MGO accumulates quickly in various tissues and play a prominent role in the pathogenesis of diabetic vascular complications [16–19]. The accumulation of MGO damages the cellular environment, leading to a state of chronic inflammation and oxidative stress that ultimately induces EC apoptosis [16, 19]. Pyroptosis is a novel form of inflammatory caspase-1-dependent programmed cell death that differs from apoptosis and other types of cell death [20–22]. The induction of pyroptosis is closely associated with the activation of the NOD-like receptor 3 (NLRP3) inflammasome [23, 24]. A study revealed that MGO stimulation promoted endothelial NLRP3 inflammasome expression in the peritoneum of mice [25]; however, the effect of MGO on EC pyroptosis has not been previously reported.

Sulforaphane (SFN), which is one of the most common plant isothiocyanates and a prominent dietary antioxidant in the human diet, is found in cruciferous vegetables such as broccoli and cabbage, and it has been shown to exhibit strong antioxidant and anti-inflammatory properties [26–29]. Previous studies have demonstrated that SFN inhibits oxidative and inflammatory progression by upregulating the expression of nuclear transcription factor erythroid 2-related factor 2 (Nrf2) and antioxidant enzymes such as heme oxygenase (HO-1) [27, 30–32]. In addition, several studies have confirmed that SFN can inhibit the activation of the NLRP3 inflammasome and alleviate pyroptosis by enhancing the Nrf2 signaling pathway [33–36]. However, whether SFN can reduce MGO-induced EC pyroptosis, the roles SFN and MGO play, and how SFN and MGO interact with each other in the context of the protective effects of SFN on the cardiovascular system remain to be determined. Targeted inhibition of related signaling pathways may be effective in the treatment of diabetic cardiovascular diseases. In the present study, we examined the protective effect of SFN against MGO-induced pyroptosis in human umbilical vein ECs (HUVECs) in vitro and the inhibitory effect of SFN on MGO-induced vascular injury and pyroptosis in vivo. We further explored the molecular mechanism, which involved reducing oxidative/inflammatory damage mediated by the NLRP3 and Nrf2/HO-1 signaling pathways.

## Materials and methods

### Chemicals, reagents, and antibodies

MGO was purchased from Sigma‒Aldrich Co. (St. Louis, MO, USA). SFN was purchased from Merck Millipore (Darmstadt, Germany). ML385 was purchased from MedChemExpress (New Jersey, USA). MCC950 was purchased from APExBIO (Houston, TX, USA). VX765 was purchased from Selleck (Shanghai, China). A Cell Counting Kit-8 (CCK-8) kit was purchased from Beyotime Biotechnology (Shanghai, China). N-acetyl cysteine (NAC) was purchased from Beyotime Biotechnology (Shanghai, China). Cyclosporin A (CsA) was purchased from Gene Operation (Ann Arbor, Michigan, USA). MGO ELISA kits were purchased from Blue Gene Biotech (Shanghai, China). The reactive oxygen species (DCFH-DA) assay kit, enhanced mitochondrial membrane potential assay kit with JC-1 (JC-1), total superoxide dismutase (SOD) assay kits, catalase (CAT) assay kits, glutathione peroxidase (GSH-Px) assay kits, and malondialdehyde (MDA) assay kits were purchased from Beyotime Biotechnology (Shanghai, China). The Omni-ECLTM Femto Light Chemiluminescence Kit and Omni-EasyTM Protein Sample Loading Buffer (Denaturing, Reducing, 5X) were purchased from EpiZyme (Shanghai, China). All other chemicals and reagents were purchased from Beyotime Biotechnology unless otherwise stated.

### Chemical structure

Searches were performed using PubChem (https://pubchem.ncbi.nlm.nih.gov) based on MGO and SFN names, CAS numbers. The search results were carefully screened and validated to ensure the accuracy and reliability of the selected structures. Download 3d models of MGO and SFN.

### Cell culture

HUVECs were obtained from the Cell Resource Center, Shanghai Institute of Biological Sciences, Chinese Academy of Sciences. HUVECs were cultured in Endothelial Cell Medium (ECM, ScienCell, Carlsbad, CA, USA) supplemented with 5% fetal bovine serum (FBS), growth factor (ECGSECGF), and penicillin/streptomycin at 37 °C with 5% CO_2_ and 95% air. Cells at passages 3–10 were used for the experiments.

### Cell viability assay

Cell viability was determined with a CCK-8 assay kit. HUVECs were seeded in 96-well plates at a density of 7 × 10^3^ cells/well, incubated for 24 h, incubated in fresh medium containing various concentrations of SFN (0, 0.5, 1, 2, 5, 10, or 20 μM) for 2 h, and then stimulated with MGO (0, 10, 20, 50, 100, or 200 μM) for 24 h. In some experiments, HUVECs were incubated with NAC (10 mM), CsA (1 μM), ML385 (20 μM), MCC950 (20 μM), VX765 (100 μM), or the vehicle control for 2 h before MGO (100 μM) treatment. Then, fresh medium containing 10 μL/100 μL CCK-8 solution was added to each well, and the plates were incubated for 1 h at 37 °C. Finally, the absorbance was measured by a Multiskan Go Microplate Reader (Molecular Devices, Waltham, MA, USA) at 450 nm.

### Western blotting

HUVECs were seeded in 6-well plates (1 × 10^5^ cells/well) under appropriate growth conditions, pretreated with SFN (0, 2, 5, or 10 μM), ML385 (20 μM), MCC950 (20 μM), VX765 (100 μM), or the vehicle control for 2 h and stimulated with MGO (100 μM) for 24 h. Total protein was obtained from HUVECs as previously described [37]. The protein concentrations were determined by a BCA protein assay kit. Equal amounts of protein lysates were separated by SDS‒PAGE and transferred onto PVDF membranes, which were subsequently blocked with 5% skim milk at room temperature for 2 h. Then, the membranes were incubated with primary antibodies against Nrf2, HO-1, NLRP3, ASC, Caspase-1, IL-1β, GSDMD, or GAPDH at 4 °C overnight. After being washed with TBST three times, the membranes were incubated with the appropriate secondary antibody (1:2000) for 1 h. The blots were developed using ECL substrate (Pierce, Rockford, IL, USA). The details of the antibodies used, including their sources and dilutions, are provided in Table 1. Band size and density analyses were performed using ImageJ software.

**Table 1:** Antibodies and dilutions used for western blotting

Antibody	Source and Cat. No	Dilution
Primary		
HO-1	Santa Cruz, sc-136960	1:1000
ASC	Santa Cruz, sc-514414	1:500
IL-1β	Cell Signaling Technology, 12242S	1:1000
Nrf2	Santa Cruz, sc-365949	1:1000
NLRP3	Cell Signaling Technology, 15101S	1:1000
Caspase-1	Cell Signaling Technology, 3866S	1:1000
GSDMD	Cell Signaling Technology, 96458S	1:1000
GAPDH	Cell Signaling Technology, 2218	1:1000
Secondary		
Horseradish peroxidase-conjugated goat anti-mouse lgG antibody raised aqainst rabbit or mouse lgG	Santa Cruz	1:2000

### Cell death assay

Pyroptotic cell death was evaluated by LDH release assays and TUNEL and caspase-1 double staining. LDH release into the cell culture supernatants and plasma were measured using an LDH assay kit according to the manufacturer’s guidelines. For TUNEL and caspase-1 double staining, the cells were fixed with 4% paraformaldehyde, incubated with permeabilization working solution, and immersed in buffer. Subsequently, the cells were incubated with TUNEL reaction mixture and blocked with BSA. The cells were then incubated with an anti-caspase-1 antibody at 4 °C overnight, followed by incubation with a secondary antibody (specific to the primary antibody species) in the dark. The nuclei were stained with DAPI for 10 min. The cells were imaged under an EVOS inverted microscope (AMG, Mill Creek, WA, USA).

### Detection of intracellular reactive oxygen species (ROS)

A ROS assay kit was used to examined the accumulation of ROS in ECs according to the manufacturer’s instructions. Briefly, HUVECs were seeded in 6-well plates and pretreated with SFN (0, 2, 5, or 10 μM), NAC (10 mM), CsA (1 μM), or the vehicle control for 2 h, followed by stimulation with MGO (100 μM) for 1 h. The treated cells were incubated with DCFH-DA (10 μM) in serum-free medium in the dark at 37 °C for 30 min, after which they were washed with PBS three times. Cellular fluorescence was measured using a Multiskan Go Microplate Reader (Molecular Devices, Waltham, MA, USA) (485/530 nm), and images were captured with a fluorescent inverted microscope.

### Measurement of intracellular MDA, SOD, CAT, and GSH-Px levels

HUVECs were seeded in 6-well plates and grown to confluence. Then, the cells were incubated with SFN (2, 5, or 10 μM), NAC (10 mM), CsA (1 μM), or the vehicle control for 2 h, followed by stimulation with MGO (100 μM) for 1 h. The protein concentration of the cell lysates was determined by a BCA protein assay. The levels of MDA, SOD, CAT, and GSH-Px were determined by the respective ELISA kits according to the manufacturer’s instructions.

### Determination of the mitochondrial membrane potential (MMP) (ΔΨm)

MMP was detected by a JC-1 assay as previously described. HUVECs were pretreated with SFN (2, 5, or 10 μM) for 2 h and subsequently stimulated with MGO (100 μM) for 1 h. The cells were washed with PBS and subsequently incubated with JC-1 (10 μg/mL) for 20 min in a humidified incubator with 5% CO_2_ at 37 °C in the dark. Then, the free probe was washed away with JC-1 buffer solution. The ratio of red-to-green fluorescence was used to evaluate changes in the MMP. The fluorescence intensities of the JC-1 monomers (490/530 nm) and aggregates (525/590 nm) were measured using a Multiskan Go Microplate Reader. Images were captured using a fluorescent inverted microscope.

### Morphological observations

Morphological abnormalities in HUVEC mitochondria were observed by transmission electron microscopy (TEM). HUVECs were fixed with 2.5% glutaraldehyde, stained with cacodylate-buffered osmium tetroxide, and embedded in epoxy resin. Sections were prepared and examined using an electron microscope (Hitachi HT7700, Tokyo, Japan).

### Animals

Male C57BL/6 mice (6 weeks old) were purchased from Chengdu Yaokang Biotechnology Co., Ltd. The mice were housed and acclimatized for 1 week before the experiments; the animals were maintained in a well-ventilated animal transit room with a 12 h light/dark cycle, a relative humidity of 60 ± 10%, and a controlled temperature of 22 °C. The mice were fed standard laboratory rodent chow and given unrestricted access to food and tap water. The protocols for animal use were reviewed and approved by the Animal Care Committee of Southwest Medical University in accordance with the Institutional Animal Care and Use Committee guidelines.

### MGO and SFN administration

MGO was administered intraperitoneally over five consecutive days each week for 7 consecutive weeks. The initial dose administered was 50 mg/kg of body weight for the first 2 weeks, followed by 60 mg/kg for 2 weeks and 75 mg/kg for the final 3 weeks. SFN was administered by subcutaneous injection at a dose of 0.5 mg/kg 5 times per week for the last 5 weeks. Both SFN and MGO were prepared with saline and stored at 4 °C in the dark. The animals were randomly selected and divided into 4 groups: the vehicle group, MGO group, SFN group, and SFN + MGO group. In addition, ear margin labeling was performed. The control group was injected with normal saline. Bodyweight and food intake were monitored throughout the study period. The mice were sacrificed by cervical dislocation, and blood and vessel samples were collected. The isolated serum was stored at – 80 °C until further analysis.

### Measurement of MGO concentrations and inflammatory factor levels

MGO concentrations in mouse serum samples were examined with ELISA kits according to the manufacturers’ instructions. The serum concentrations of the inflammatory cytokines IL-1β and IL-18 were measured with ELISA kits according to the manufacturer’s instructions.

### H&E staining

The vessels were fixed with 4% paraformaldehyde for 24 h. Then, the vessels were dehydrated, cleared, embedded in paraffin and cut into paraffin sections. The sections were deparaffinized with xylene and rehydrated using gradient ethanol. After being stained with hematoxylin, the sections were stained with 0.5% eosin, dehydrated with ethanol and treated with xylene. The sections were observed under a microscope.

### Immunohistochemical analysis and quantification

The aorta samples were fixed in 4% paraformaldehyde, embedded in paraffin, and cut into sections for immunohistochemical staining. The sections were dewaxed in xylene (100%), hydrated in a graded alcohol series (100, 90, 75, and 50%) and incubated with 3% H2O2 for 15 min at room temperature to quench endogenous peroxidase activity. Subsequently, the vessels were subjected to antigen retrieval by incubation in sodium citrate buffer for 10 min at 90 °C. The slides were blocked with normal goat serum for 15 min, after which the sections were incubated with primary antibodies against NLRP3 (Thermo Fisher Scientific, 1:500, Cat. No.: MA5-32,255), Caspase-1 (Santa Cruz, 1:200, Cat. No.: sc-392736), GSDMD (Abcam, 1:1000, Cat. No.: ab219800), IL-1β (Cell Signaling Technology, 1:200, Cat. No.: 12242S), and Nrf2 (Thermo Fisher Scientific, 1:500, Cat. No.: PA5-27,882) at 4 °C overnight. After being washed three times with PBS, the secondary antibody (Beyotime Biotechnology, China) was added and incubated for 1 h at room temperature. Hematoxylin was used for background counterstaining. For quantification, the semiquantitative immunohistochemical score was determined by examining at least five random fields per section under × 100 magnification, and digital image analysis was performed with ImageJ software.

### Statistical analysis

The data were analyzed with GraphPad Prism 8.0.1 software (GraphPad Software, San Diego, CA, USA) and are presented as the mean ± standard deviation (SD) of at least three independent experiments. Statistical analyses were performed by Student’s t test and one-way ANOVA followed by the Bonferroni post hoc correction. *P* < 0.05 was considered to indicate statistical significance.

## Results

### Effects of SFN on MGO-injured HUVEC viability and pyroptosis

**Fig. 1:**
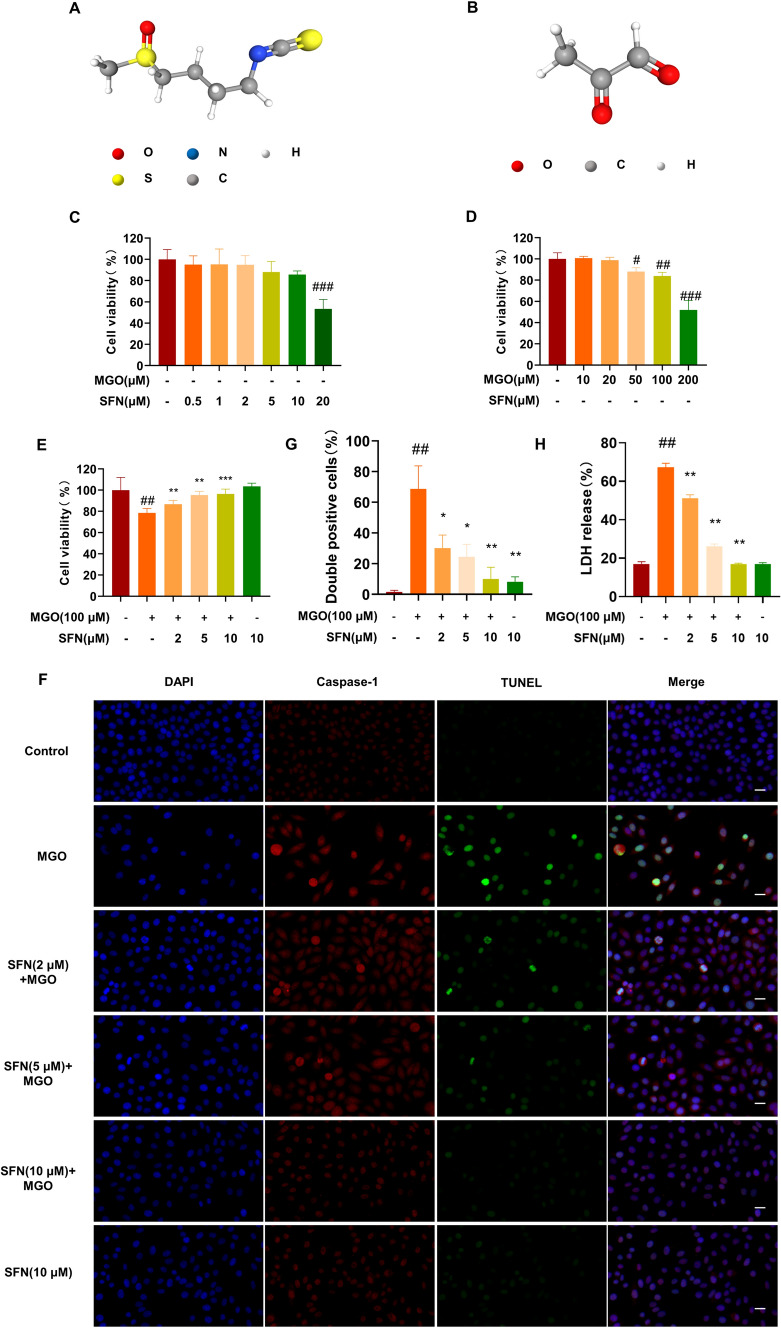
SFN suppresses MGO-induced pyroptosis **A**, **B** The chemical structures of SFN and MGO. **C** HUVECs were treated with SFN (0–20 μM, 24 h), and the CCK-8 assay was subsequently performed to determine cell viability. **D** Cells were treated with MGO (0–200 μM) for 24 h. Cell viability was assessed by the CCK-8 assay. **E** ECs were pretreated with SFN (0, 2, 5, or 10 μM) for 2 h followed by MGO (100 μM) exposure for 24 h. Cell viability was assessed by the CCK-8 assay. **F**, **G** HUVECs were treated with SFN (0, 2, 5, or 10 μM) for 2 h, followed by MGO (100 μM) treatment for 24 h. Pyroptosis was examined by TUNEL (green) and caspase-1 (red) double-positive staining. The nuclei were stained blue with DAPI. Representative images of pyroptotic cells are shown. The scale bar represents 20 μm. **H** Pyroptosis was examined by measuring LDH release (%) in the cell culture supernatant. The values are presented as the means ± SD from three independent experiments. ^#^*P* < 0.05 vs. Ctrl, ^##^*P* < 0.01 vs. Ctrl, ^###^*P* < 0.001 vs. Ctrl, ^*^P < 0.1 vs. MGO, ^**^P < 0.01 vs. MGO, ^***^P < 0.001 vs. MGO

We first examined the effects of SFN (Fig. 1A) on the viability of MGO-injured HUVECs (Fig. 1B) by using the CCK-8 assay. As shown in Fig. 1C, exposing HUVECs to 20 μM SFN for 24 h significantly reduced cell viability, but no obvious side effects were observed after treatment with 0.5, 1, 2, 5, or 10 μM SFN. Furthermore, we found that treatment with 50, 100, or 200 μM MGO for 24 h significantly inhibited the viability of HUVECs (Fig. 1D), which was in consistent the findings of previous studies. A shown in Fig. 1E, pretreatment with 2, 5, or 10 μM SFN for 2 h dose-dependently reversed MGO-mediated inhibition of HUVEC viability.

To evaluate the effect of SFN on MGO-induced pyroptosis, HUVECs were pretreated with various concentrations (2, 5, or 10 μM) of SFN and then stimulated with MGO (100 μM). Then, caspase-1 and TUNEL double staining were performed. Treatment with MGO markedly activated caspase-1 activity and increased the number of TUNEL-positive cells relative to those in the untreated control group. Moreover, the increase in pyroptosis was significantly suppressed by SFN, particularly at a concentration of 10 μM (Fig. 1F, G). Lactate dehydrogenase (LDH) release was used to examine cell death. MGO treatment significantly increased LDH release in the cell culture supernatant (Fig. 1H), and these effects were abrogated by SFN treatment in a dose-dependent manner. These results indicate that SFN can inhibit MGO-induced pyroptosis in HUVECs.

### SFN prevents MGO-induced NLRP3 inflammasome activation and ameliorates impairment of the Nrf2/HO-1 pathway

**Fig. 2:**
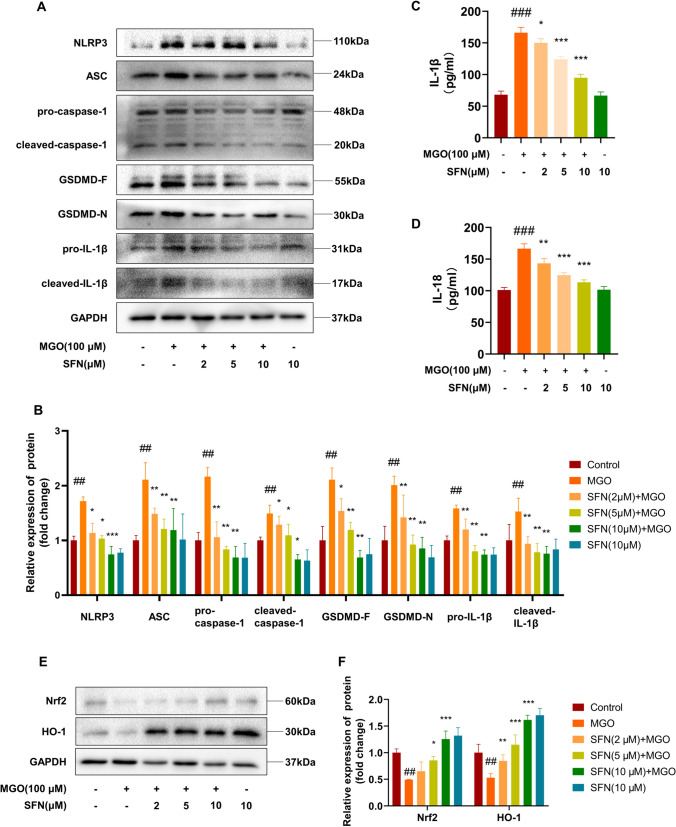
SFN prevents MGO-mediated NLRP3 inflammasome activation and suppresses the impairment of the Nrf2/HO-1 pathway HUVECs were treated with SFN (0, 2, 5, or 10 μM) for 2 h, followed by MGO (100 μM) treatment for 24 h. **A**, **B** The effects of SFN on MGO-induced changes in the inflammasome-related proteins NLRP3, ASC, pro-caspase-1, and cleaved caspase-1, as well as the pyroptosis-related proteins GSDMD-F, GSDMD-N, pro-IL-1β, and cleaved-IL-1β, were examined by western blotting. GAPDH was used as the loading control. **C**, **D** The levels of IL-1β and IL-18 in the supernatants were determined by ELISA. **E**, **F** The levels of the oxidation-related proteins Nrf2 and HO-1 were investigated by western blotting. All the graphs correspond to the blots above and represent the densitometric analyses of three independent experiments; the data are expressed as the means ± SD. ^##^*P* < 0.01 vs. Control, ^###^*P* < 0.001 vs. Ctrl, ^*^P < 0.05 vs. MGO, ^**^P < 0.01 vs. MGO, ^***^P < 0.001 vs. MGO

Pyroptosis can be mediated by the effector molecule GSDMD [38, 39]. In the present study, SFN pretreatment significantly inhibited the increases in the levels of the full-length GSDMD and the GSDMD N-terminus in MGO-treated cells (Fig. 2A, B). NLRP3-mediated inflammatory activation is also involved in the cleavage of full-length GSDMD to generate the GSDMD N-terminus, which is the effector molecule of pyroptosis [40, 41]. To determine the effect of SFN on MGO-induced changes in NLRP3 inflammasome activation, the protein levels of NLRP3, ASC, pro-caspase-1, and pro-IL-1β were examined by western blotting. As shown in Fig. 2A, B, MGO significantly increased the levels of NLRP3, ASC, pro-caspase-1, and pro-IL-1β, while SFN treatment significantly inhibited the production of NLRP3 inflammasome components. Consistently, the mature forms of caspase-1 and IL-1β were measured in cell extracts after exposure to 100 μM MGO, and SFN reduced the levels of these proteins. As shown in Fig. 2C and D, ELISA showed that IL-1β and IL-18 levels were consistent with these findings. These data demonstrate that SFN can inhibit MGO-induced pyroptosis and NLRP3 inflammasome activation in HUVECs. The Nrf2/HO-1 pathway plays a pivotal role in the survival of certain cell types and is involved in the adaptive response to oxidative stress and inflammatory responses [42–44]. Previous studies have also suggested that SFN-mediated antioxidant and anti-inflammatory effects might involve the activation of Nrf2 and HO-1 [27, 45]. To better understand the molecular mechanisms by which SFN affects MGO-induced pyroptosis, the expression levels of Nrf2 and HO-1 were examined by western blotting. The data showed that MGO significantly reduced the expression levels of Nrf2 and HO-1 in HUVECs (Fig. 2E, F). Furthermore, preincubation with various concentrations of SFN abrogated the MGO-mediated reductions in Nrf2/HO-1 protein levels (Fig. 2 E, F). These results linked MGO-induced pyroptosis and the Nrf2/HO-1 signaling pathway, which was modulated by SFN.

### SFN suppresses MGO-induced intracellular ROS generation

**Fig. 3:**
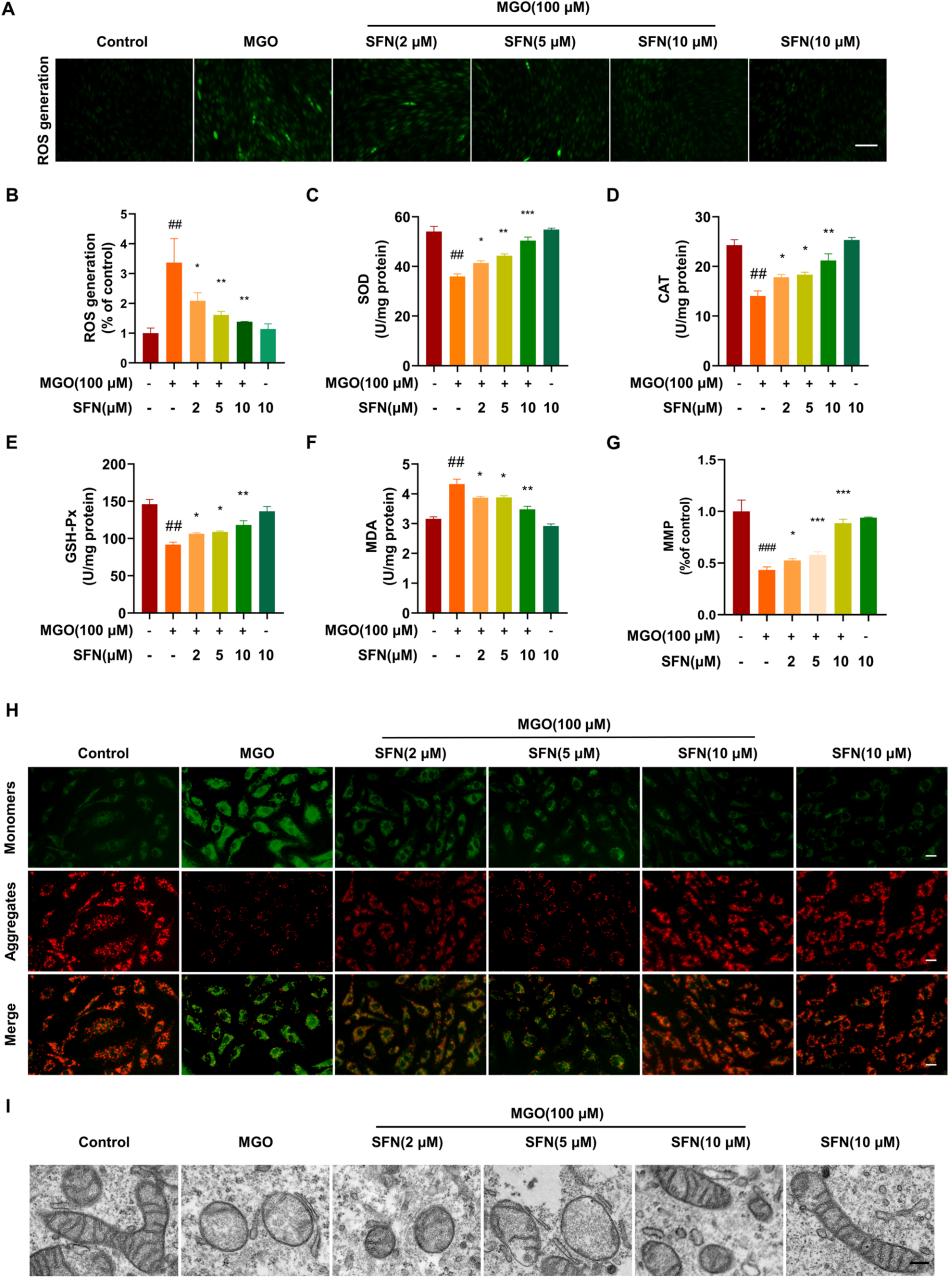
SFN suppresses MGO-induced intracellular ROS generation and mitochondrial damage HUVECs were treated with SFN (0, 2, 5, or 10 μM) for 2 h and then exposed to MGO (100 μM) for 1 h. **A**, **B** The cells were stained with DCFH-DA, and the fluorescence intensity was measured at 488/525 nm using a microplate reader. Scale bar, 250 nm. (C-F) The levels of SOD, CAT, GSH-Px and MDA were measured with the indicated ELISA kits. **G**, **H** The MMP was assessed with the JC-1 probe. The fluorescence intensities of JC-1 monomers (490/530 nm) and JC-1 aggregates (525/590 nm) were measured using a microplate reader. The ratio of JC-1 aggregates/JC-1 monomers was calculated. Scale bar, 50 μm. **I** Ultrastructural alterations in mitochondria were detected by TEM. Scale bars: 200 nm. The data are shown as the means ± SD of three independent experiments. ^##^*P* < 0.01 vs. Control, ^###^*P* < 0.001 vs. Control, ^*^P < 0.05 vs. MGO, ^**^P < 0.01 vs. MGO, ^***^P < 0.001 vs. MGO

Oxidative stress is caused by an imbalance between excessive and/or sustained increases in ROS production and deficiencies in antioxidant defenses [46–48]. Increased intracellular ROS production has been shown to be involved in regulating pyroptosis [49, 50]. To determine whether SFN can attenuate MGO-induced ROS production, DCFH-DA assays were performed to examine intracellular ROS levels. As shown in Fig. 3A, B, MGO significantly increased ROS production in HUVECs. Treatment with SFN suppressed ROS production in HUVECs in a dose-dependent manner, especially in the presence of 10 μM SFN. Furthermore, the activity of antioxidant enzymes, including SOD, CAT, and GSH-Px, was significantly decreased by MGO. Treatment with SFN markedly ameliorated the MGO-induced decrease in antioxidant enzyme activity in a dose-dependent manner, particularly in response to 10 μM SFN (Fig. 3C–E). MDA levels were also measured. Treatment with MGO significantly increased MDA levels, but this change was reversed by SFN pretreatment (Fig. 3F). These results suggest that SFN prevents MGO-induced oxidative stress.

### SFN prevents MGO-induced mitochondrial damage

Pyroptotic cell death is linked to mitochondrial dysfunction, which is characterized by the MMP [50, 51]. Previous studies have shown that MGO induces the opening of permeability transition pores (PTPs) and thus reduces MMP levels [16, 52]. As shown in Fig. 3G and H, our data showed that MGO markedly reduced MMP levels compared to those in the control group, suggesting that the MMP was depolarized. Dramatically, preincubation with SFN dose-dependently inhibited MGO-induced MMP depolarization. Furthermore, mitochondrial morphology was analyzed and imaged using TEM. Normal mitochondria with preserved membranes and cristae were observed in HUVECs in the control group (Fig. 3I). After the cells were treated with MGO (100 μM) for 24 h, damaged mitochondria were observed, and the outer membranes and cristae showed the loss of ultrastructural integrity (Fig. 3I). Notably, treatment with SFN protected against mitochondrial morphological alterations, suggesting that SFN can prevent MGO-induced mitochondrial damage.

### Effects of NAC and CsA on MGO-induced HUVEC pyroptosis and ROS generation

**Fig. 4:**
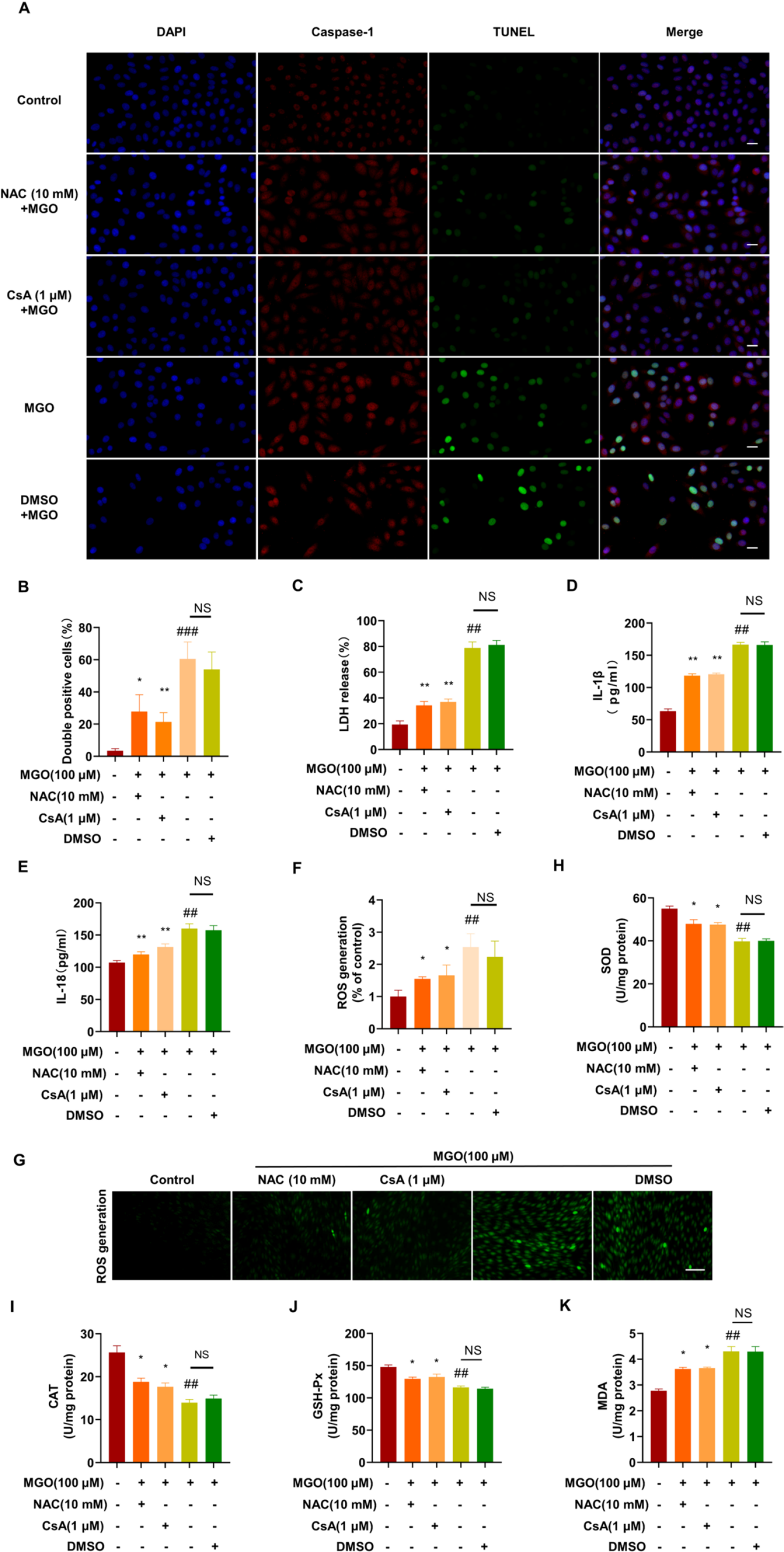
Effects of NAC and CsA on MGO-induced HUVEC pyroptosis and ROS generation HUVECs were pretreated with NAC (10 mM) and CsA (1 μM) for 2 h and then stimulated with MGO for 24 h. **A**, **B** Pyroptosis was examined by TUNEL (green) and caspase-1 (red) double-positive staining. The nuclei were stained blue with DAPI. Representative images of pyroptotic cells are shown. The scale bar represents 20 μm. **C** Pyroptotic cell death was determined by measuring LDH release. **D**, **E** ELISA analysis of IL-1β and IL-18 in the supernatants of HUVECs subjected to the different treatments. **F**, **G** The cells were stained with DCFH-DA, and the fluorescence intensity was measured at 488/525 nm using a microplate reader. Scale bar, 250 nm. **H**–**K** The levels of SOD, CAT, GSH-Px, and MDA were measured with the appropriate kits according to the manufacturer’s instructions. The values (mean ± SD from three independent experiments) are relative to the control and are expressed as fold changes. ^*##*^*P* < 0.01 vs. Control, ^*###*^*P* < 0.001 vs. Control, ^*^P < 0.05 vs. MGO, ^**^P < 0.01 vs. MGO, *NS*: not significant

To confirm whether the inhibition of mitochondrial dysfunction is associated with antipyroptotic effects, HUVECs were pretreated with CsA (1 μM, an inhibitor of the mammalian PTP) and stimulated with MGO, after which pyroptosis was measured. As shown in Fig. 4A, CsA significantly inhibited MGO-induced pyroptosis, indicating that the protective effects of SFN against pyroptosis involve inhibiting mitochondrial dysfunction. Furthermore, to investigate the effects of antioxidants on MGO-induced pyroptosis and ROS generation, HUVECs were pretreated with the ROS scavenger NAC (10 mM) and then stimulated with MGO. NAC pretreatment reduced the number of TUNEL and Caspase-1 double-positive cells (Fig. 4A, B) and LDH activity in MGO-treated ECs (Fig. 4C). NAC treatment significantly decreased the IL-1β and IL-18 concentrations in the cell culture supernatant (Fig. 4D, E). Moreover, as shown in Fig. 4F, G, MGO-induced ROS production in HUVECs was markedly decreased in the NAC group. In addition, NAC significantly inhibited the increase in MDA levels induced by MGO (Fig. 4H). Conversely, NAC significantly ameliorated the inhibitory effects on the activity of antioxidant enzymes (SOD, CAT, and GSH-Px) induced by treatment with MGO (Fig. 4I–K). These results indicated that NAC significantly inhibited MGO-induced HUVEC pyroptosis and ROS generation.

### An Nrf2 inhibitor attenuates the protective effect of SFN against MGO-induced pyroptosis

**Fig. 5:**
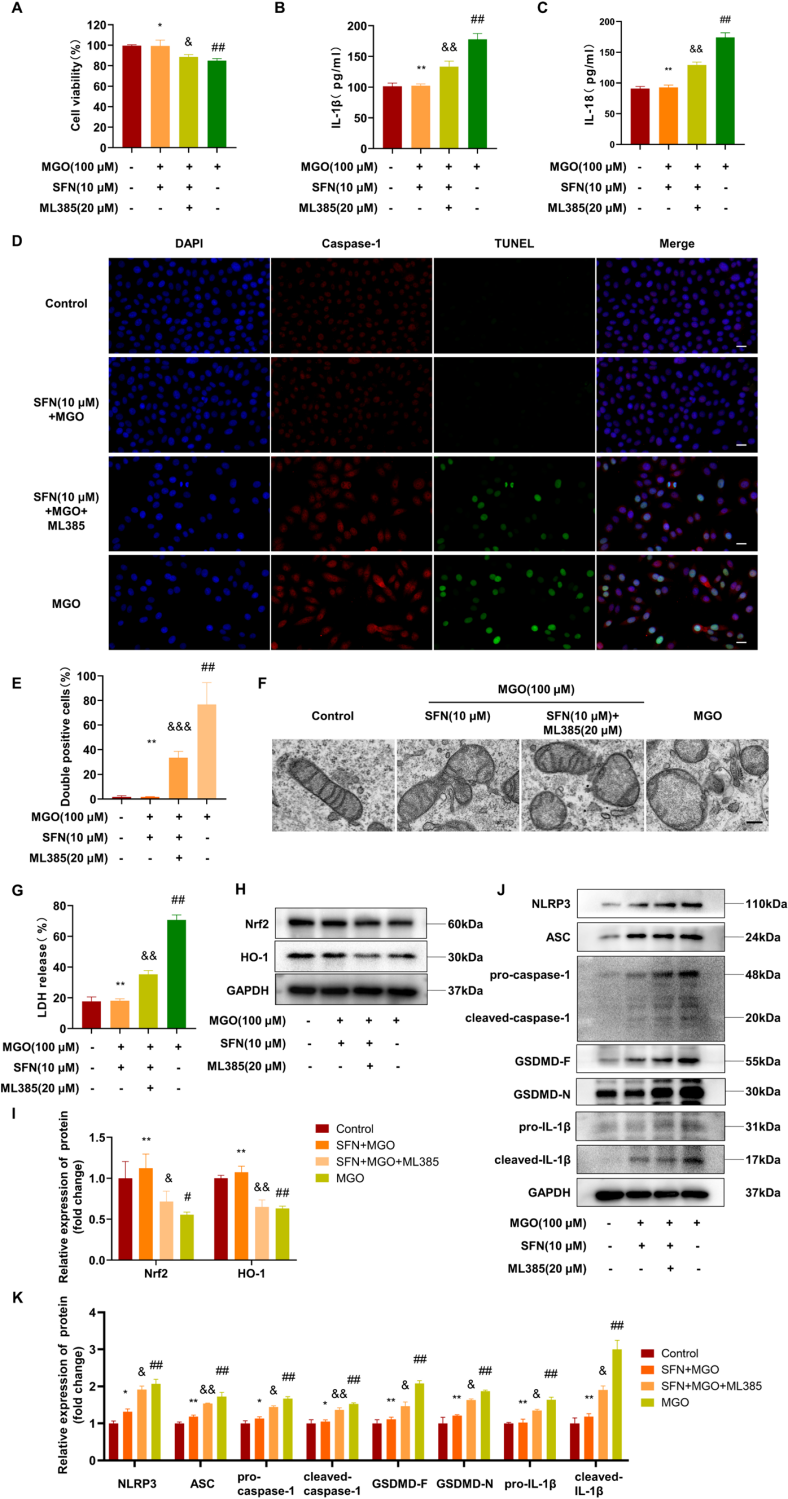
The Nrf2 inhibitor attenuates the protective effect of SFN against MGO-induced pyroptosis HUVECs were pretreated with SFN (10 μM), ML385 (20 μM, an Nrf2 inhibitor), or the vehicle control for 2 h and subsequently stimulated with MGO for 24 h. **A** Cell viability was determined by a CCK-8 assay. **B**, **C** ELISA analysis of IL-1β and IL-18 in the supernatants of HUVECs subjected to different treatments. **D**, **E** Pyroptosis was examined by TUNEL (green) and caspase-1 (red) double-positive staining. The nuclei were stained blue with DAPI. Representative images of pyroptotic cells are shown. Scale bar represents 20 μm. **F** Ultrastructural alterations in mitochondria were examined by TEM. Scale bars: 200 nm. **G** Pyroptosis was measured by measuring LDH release (%) in the cell culture supernatant. **H**, **I** Representative western blot analysis of total cell lysates with antibodies against Nrf2 and HO-1; the proteins were quantified by densitometry and are presented as ratios relative to GAPDH. **J**, **K** The protein expression levels of NLRP3, ASC, pro-caspase-1 and cleaved caspase-1 were measured by western blotting and quantified by ImageJ software. The expression of GSDMD-F, GSDMD-N, pro-IL-1β and cleaved IL-1β in the cell lysate was examined immunoblot assays and quantified by normalization to the control group. The values (mean ± SD from three independent experiments) are relative to the control and are expressed as fold changes. ^#^*P* < 0.05 vs. Control, ^##^*P* < 0.01 vs. Control, ^*^P < 0.05 vs. MGO, ^**^P < 0.01 vs. MGO, ^&^*P* < 0.05 vs. SFN + MGO, ^&&^*P* < 0.01 vs. SFN + MGO, ^&&&^*P* < 0.001 vs. SFN + MGO

To investigate whether Nrf2 signaling is involved in the protective effect of SFN, we pretreated HUVECs with SFN (10 μM), ML385 (20 μM, an Nrf2 inhibitor), or the vehicle control for 2 h and subsequently stimulated the cells with MGO for 24 h. As shown in Fig. 5A and S1A, the CCK-8 assay revealed that ML385 significantly attenuated the protective effects of SFN against MGO-induced pyroptosis in the absence of toxic effects on cells. Pretreatment with 20 μM ML385 significantly increased the release of active IL-1β and IL-18 by HUVECs treated with SFN plus MGO (Fig. 5B, C). TUNEL and Caspase-1 double staining also showed similar results (Fig. 5D, E). Furthermore, the release of LDH was markedly increased by ML385 treatment (Fig. 5G). Next, we investigated the effect of ML385 on mitochondrial morphological alterations. Figure 5F shows that ML385 counteracted the protective effect of SFN on mitochondrial morphology. Additionally, the levels of Nrf2 and HO-1 were evaluated by western blotting after treatment with the inhibitor ML385. Pretreatment with SFN increased the levels of Nrf2 and HO-1 compared to those in the MGO group. Conversely, treatment with ML385 antagonized the antioxidant effects of SFN and decreased Nrf2 and HO-1 expression in the presence of MGO and SFN (Fig. 5H-I). We also found that ML385 treatment increased the protein levels of NLRP3, ASC, pro-caspase-1, and pro-IL-1β and promoted the maturation of caspase-1 and IL-1β (Fig. 5J, K). Moreover, we found that SFN significantly decreased the expression of GSDMD and the cleavage of GSDMD-N in MGO-induced HUVECs, and ML385 reversed these effects (Fig. 5J-K). These results indicated that ML385 markedly attenuated the suppressive effects of SFN on the NLRP3 inflammasome signaling pathway and pyroptosis.

### SFN suppresses MGO-induced pyroptosis by inhibiting NLRP3 inflammasome activation

**Fig. 6:**
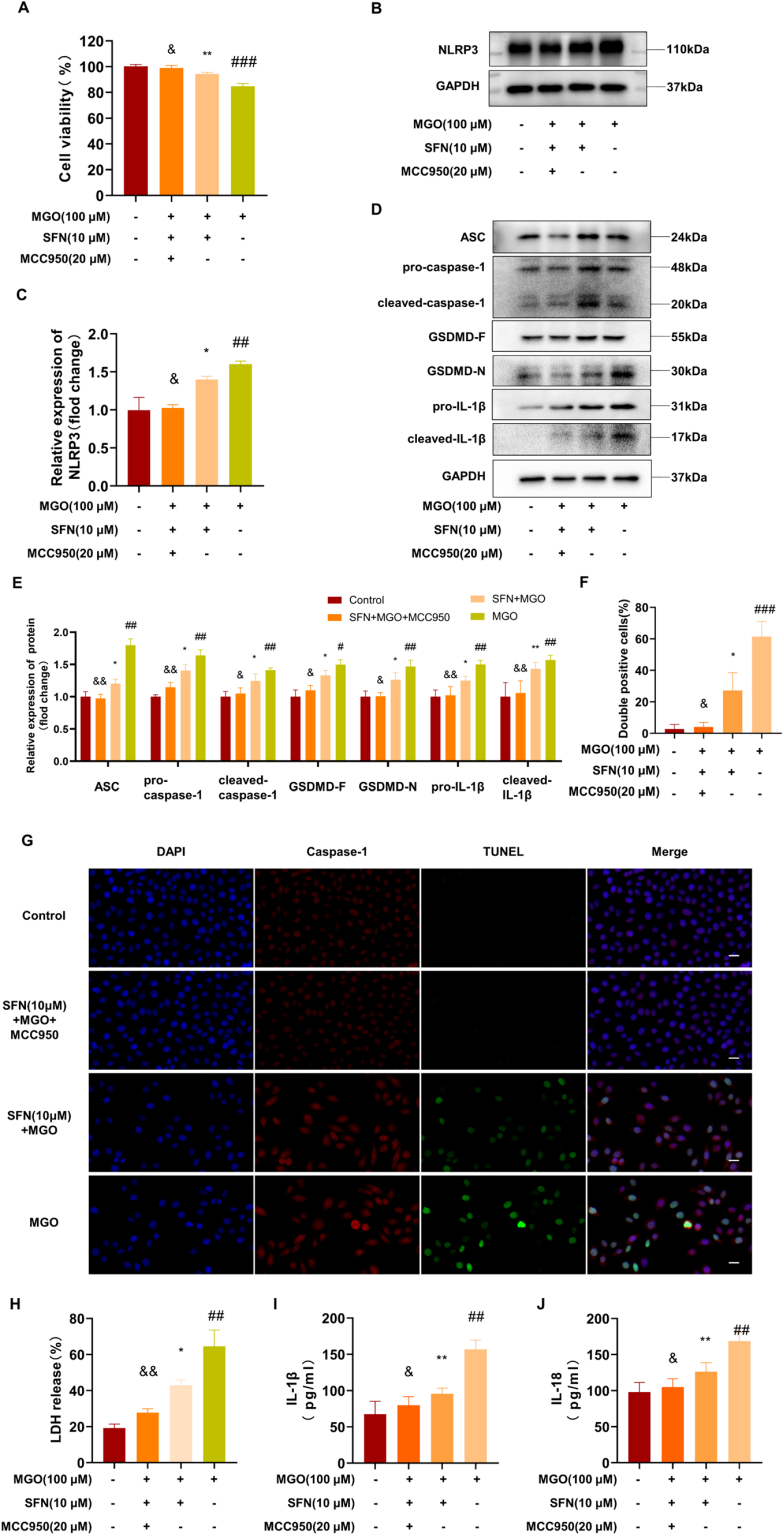
SFN suppresses MGO-induced pyroptosis by inhibiting NLRP3 inflammasome activation HUVECs were pretreated with SFN (10 μM), MCC950 (20 μM, the NLRP3 inhibitor), or the vehicle control for 2 h and then stimulated with 100 μM MGO for 24 h. **A** Then, the CCK-8 assay was performed to examine cell viability. (B-C) NLRP3 protein expression in ECs was determined by western blotting and quantified by Image J software. **D**, **E** The protein levels of ASC, pro-caspase-1 and cleaved caspase-1 were examined by western blotting. The levels of the pyroptotic proteins GSDMD-F, GSDMD-N, pro-IL-1β and cleaved-IL-1β were analyzed by western blotting. GAPDH was used as an internal control. **F**, **G** Pyroptosis was examined by TUNEL (green) and caspase-1 (red) double-positive staining. The nuclei were stained blue with DAPI. Representative images of pyroptotic cells are shown. Scale bar represents 20 μm. **H** Pyroptosis was measured by measuring LDH release (%) in the cell culture supernatant. **I**, **J** ELISA analysis of IL-1β and IL-18 in the supernatants of HUVECs subjected to different treatments. The data are shown as the mean ± SD of at least three independent experiments. ^#^*P* < 0.05 vs. Control, ^##^*P* < 0.01 vs. Control, ^###^*P* < 0.001 vs. Control, ^*^P < 0.05 vs. MGO, ^**^P < 0.01 vs. MGO, ^&^*P* < 0.05 vs. SFN + MGO, ^&&^*P* < 0.01 vs. SFN + MGO

Furthermore, to determine the importance of the NLRP3 inflammasome pathway in MGO-induced pyroptosis and investigate whether this effect was suppressed by SFN, we pretreated HUVECs with SFN (10 μM), MCC950 (20 μM, the NLRP3 inhibitor), or the vehicle control for 2 h and subsequently stimulated the cells with MGO for 24 h. The decrease in HUVEC viability induced by MGO was reversed by SFN. MCC950 had no damaging effect on cell viability (Fig. S1B) and markedly strengthened the protective effects of SFN on HUVEC viability (Fig. 6A). Moreover, the results showed that MCC950 decreased the protein levels of NLRP3, ASC, pro-caspase-1, pro-IL-1β, and GSDMD and inhibited the maturation of caspase-1, IL-1β, and GSDMD-N (Fig. 6B–E). Furthermore, MCC950 enhanced the ability of SFN to reduce HUVEC pyroptosis, as evidenced by decreases in the numbers of TUNEL- and caspase-1-double-positive cells (Fig. 6F, G). MCC950 also abrogated the release of LDH, IL-1β, and IL-18, indicating that SFN inhibited cell lysis and pyroptotic cell death (Fig. 6H–J). These data suggest that the protective effects of SFN against MGO-induced pyroptosis are partly mediated through the Nrf2/HO-1 and NLRP3 inflammasome signaling pathways.

### Caspase-1 is involved in ECs pyroptosis induced by MGO

**Fig. 7:**
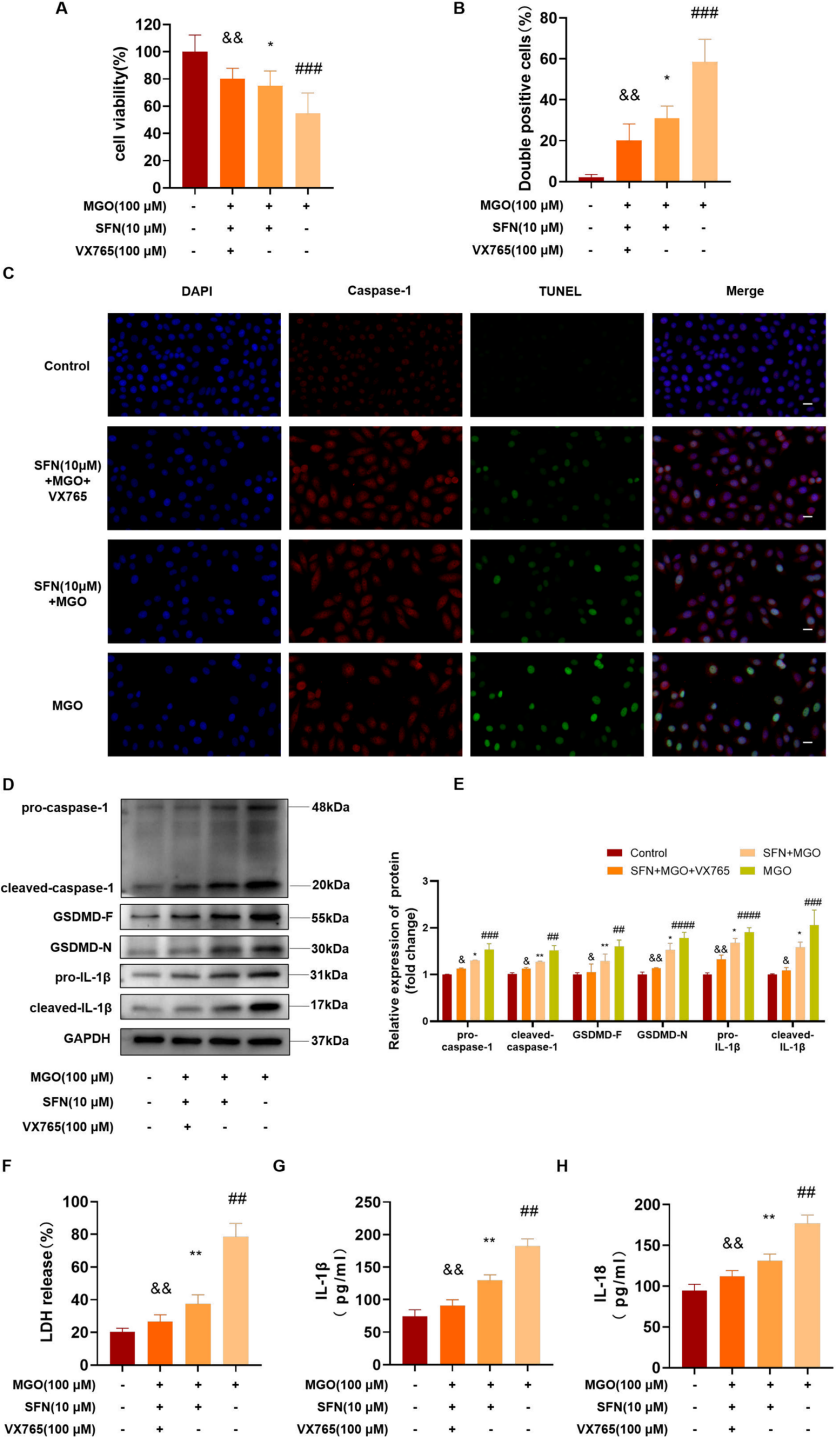
Caspase-1 is involved in endothelial cells pyroptosis induced by MGO. HUVECs were pretreated with SFN (10 μM), VX765 (100 μM, a caspase-1 inhibitor), or the vehicle control for 2 h and then stimulated with 100 μM MGO for 24 h. **A** The CCK-8 assay was performed to examine cell viability. **B**, **C** Pyroptosis was examined by TUNEL (green) and caspase-1 (red) double-positive staining. The nuclei were stained blue with DAPI. Representative images of pyroptotic cells are shown. Scale bar represents 20 μm. **D**, **E** The protein levels of pro-caspase-1, cleaved caspase-1 were examined by western blotting. The levels of the pyroptotic proteins GSDMD-F, GSDMD-N, pro-IL-1β and cleaved-IL-1β were analyzed by western blotting and quantified by Image J software. GAPDH was used as an internal control. **F** Pyroptosis was measured by measuring LDH release (%) in the cell culture supernatant. **G**, **H** ELISA analysis of IL-1β and IL-18 in the supernatants of HUVECs subjected to different treatments. The data are shown as the mean ± SD of at least three independent experiments. ^##^*P* < 0.01 vs. Control, ^###^*P* < 0.001 vs. Control, ^####^*P* < 0.0001 vs. Control, ^*^P < 0.05 vs. MGO, ^**^P < 0.01 vs. MGO, ^&^*P* < 0.05 vs. SFN + MGO, ^&&^*P* < 0.01 vs. SFN + MGO

To determine the importance of caspase-1 in MGO-induced pyroptosis and investigate whether this effect was suppressed by SFN, we pretreated HUVECs with SFN (10 μM), VX765 (100 μM, the caspase-1 inhibitor), and the vehicle control for 2 h, followed by stimulation with MGO for 24 h. As shown in Fig. 7A and Fig. S1C, VX765 had no effect on cell viability but significantly reinforced the protective effects of SFN against the reduction in cell viability induced by MGO. TUNEL and caspase-1 double staining also analogous results (Fig. 7B, C). In addition, the levels of caspase-1, GSDMD, and IL-1β were evaluated with the inhibitor VX765 by western blotting. As shown in Fig. 7D, E, pretreatment with SFN decreased the levels of pro-caspase-1, GSDMD-F, and IL-1β compared to MGO treatment. Moreover, treatment with VX765 enhanced some of the anti-inflammatory effects of SFN and decreased cleaved-caspase-1, GSDMD-N and cleaved-IL-1β levels based on the combined use of MGO and SFN. Next, we investigated the effect of VX765 on LDH release, IL-1β, and IL-18 levels in serum. Figure 7F, H revealed that VX765 strengthened the protective effect of SFN on MGO-induced pyroptosis. As expected, these data confirmed that stimulation with VX765 markedly attenuated the protective effects of SFN on caspase-1-dependent pyroptosis.

### Effects of SFN and MGO administration on physiological changes, oxidative indices, and inflammatory factors in mice

**Fig. 8:**
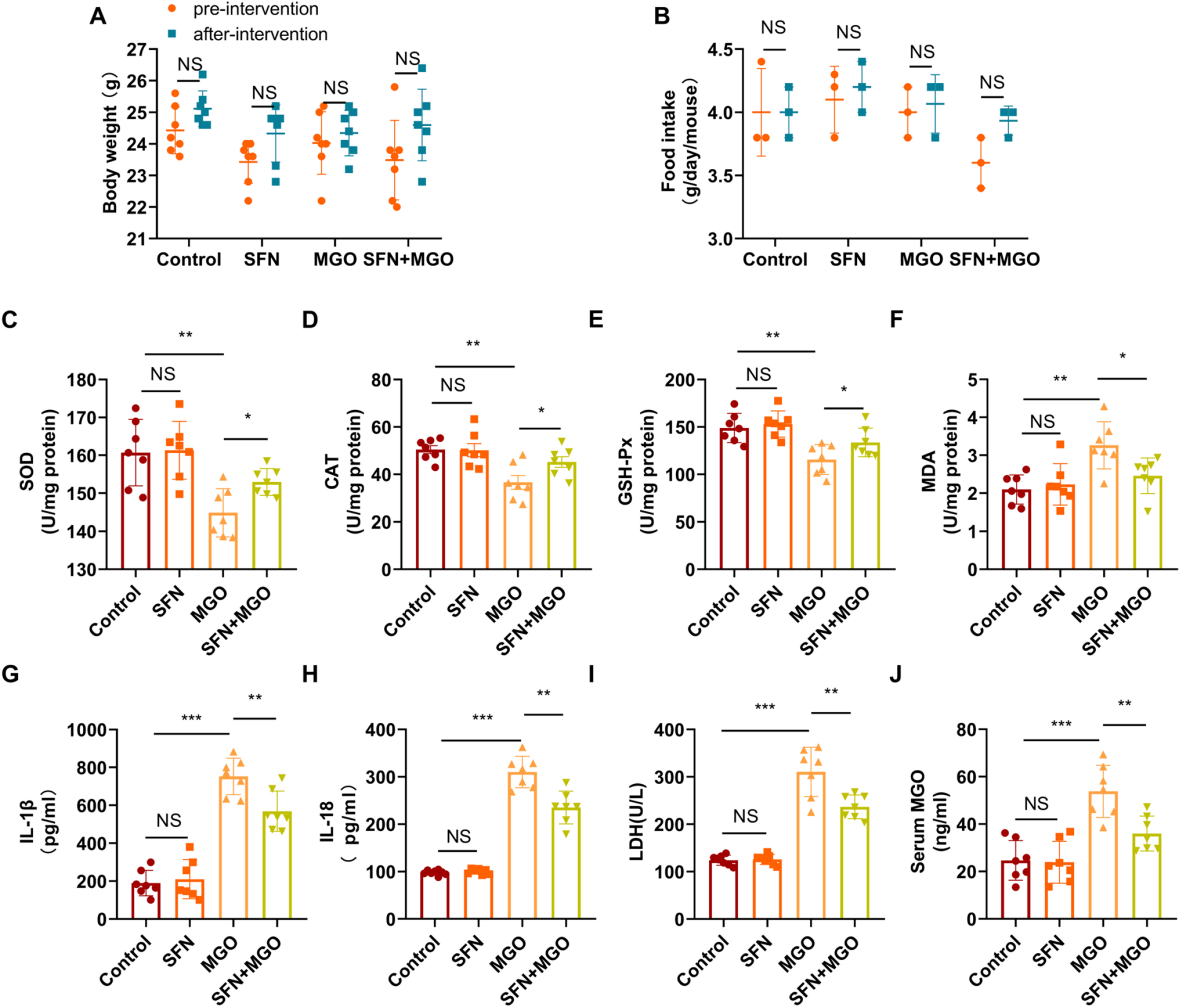
Effects of SFN and MGO on physiological changes, oxidative indices, and inflammatory factors in mice C57BL/6 mice were treated with MGO and SFN, and physiological and biochemical characteristics were analyzed. At the end of the experiment, blood samples were collected and analyzed. **A** Bodyweight. **B** Food intake. **C**–**E** The levels of SOD, CAT, and GSH-Px were measured with the appropriate kits according to the manufacturer’s instructions. **F** MDA levels was measured. **G**–**I** Changes in the proinflammatory cytokines IL-1β and IL-18 and LDH release were examined. **J** An MGO ELISA Kit was used to measure MGO levels in serum. The data are presented as the mean ± SD; n ≥ 5 for each group. ^*^P < 0.05, ^**^P < 0.01, ^***^P < 0.001, *NS*: not significant

To determine whether SFN influences MGO-induced vascular injury in vivo, C57BL/6 mice were treated with MGO and SFN. Interestingly, there were no significant changes in bodyweight or food intake in drug-treated mice compared with vehicle-treated mice (Fig. 8A, B). As shown in Fig. 8C–F, MGO significantly decreased the levels of SOD, CAT, and GSH-Px and increased MDA, but these changes were partially reversed by SFN pretreatment. These results are consistent with those obtained from the in vitro studies (Fig. 3C–F), suggesting that SFN suppresses MGO-induced oxidative stress in vivo and in vitro.

Dynamic changes in the levels of the proinflammatory cytokines IL-1β and IL-18 were investigated. As shown in Fig. 8G, H, MGO markedly increased IL-1β and IL-18 levels, and these effects were markedly inhibited by SFN. Similar effects on LDH release were confirmed, as shown in Fig. 8I. LDH release was also notably lower after SFN treatment than after MGO treatment. To determine the absorption of MGO in mice, serum concentrations of MGO were determined. MGO serum levels were increased ∼twofold compared with those in vehicle-treated mice (Fig. 8J). These data indicate the anti-inflammatory activity of SFN against MGO-induced inflammation in vivo.

### SFN prevents MGO-induced pyroptosis by regulating the Nrf2/HO-1 and NLRP3 inflammasome pathways in vivo

**Fig. 9:**
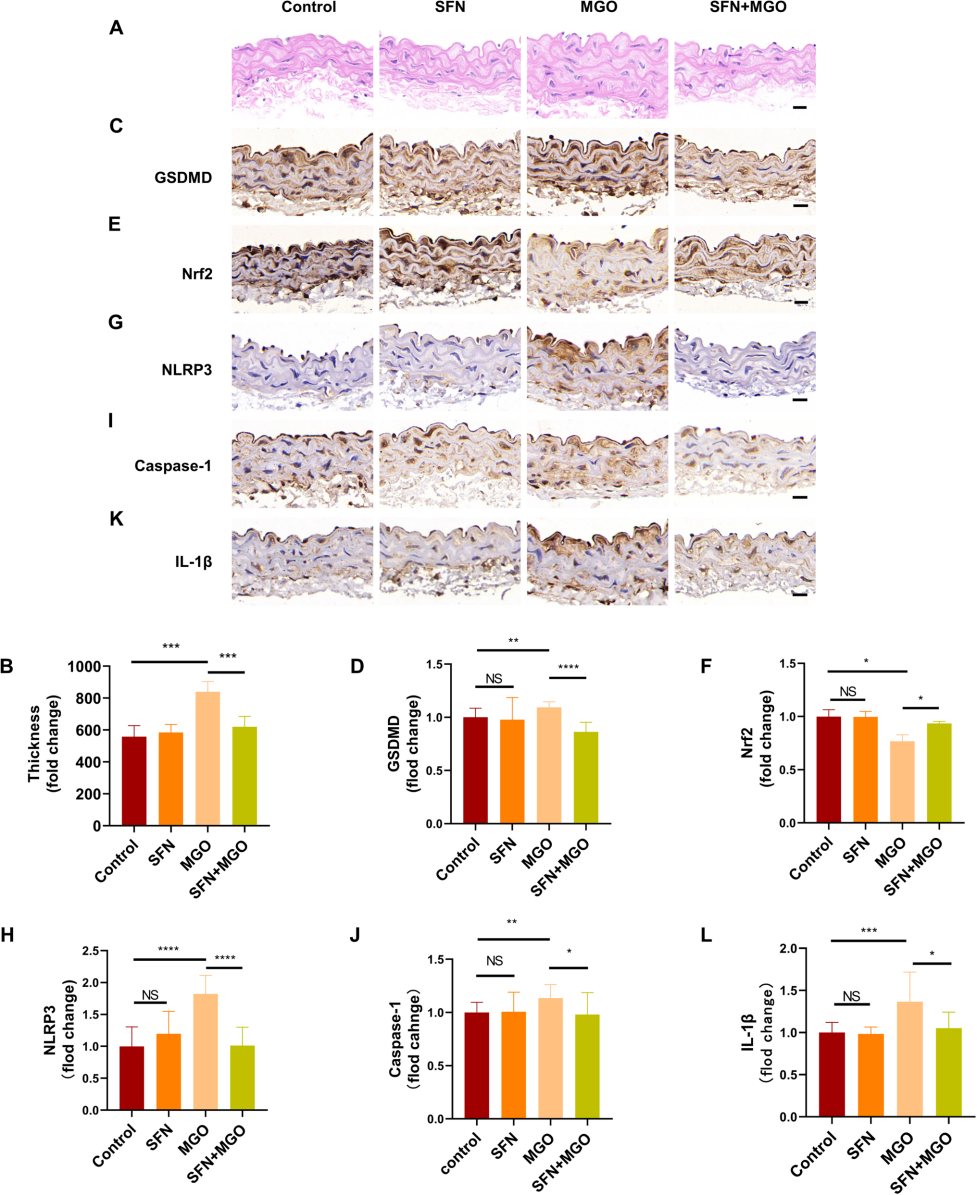
SFN prevents MGO-induced pyroptosis by regulating the Nrf2/HO-1 and NLRP3 inflammasome pathways in vivo C57BL/6 mice were treated with MGO and SFN, after which the aortas were collected for biochemical parameter analysis. **A**, **B** Histological changes in the aortas were evaluated by H&E staining. **C**, **D** GSDMD expression was evaluated by immunohistochemical staining. **E**, **F** The oxidative factor Nrf2 was examined by immunohistochemical staining. **G**–**L** The inflammatory factors NLRP3, caspase-1 and IL-1β were evaluated by immunohistochemical staining. The data are presented as the mean ± SD; n ≥ 5 for each group. *P < 0.05, **P < 0.01, ***P < 0.001, NS: not significant. Bar = 20 μm

The effect of SFN on MGO-induced pyroptosis was examined in vivo by subcutaneously administering SFN to C57BL/6 mice. Aortas were examined pathologically by H&E staining (Fig. 9A, B), and the results revealed an increase in aortic thickening after stimulation with MGO for 7 weeks. Treatment with SFN prevented MGO-induced pathological alterations. To examine the preventive effect of SFN on MGO-induced pyroptosis, immunohistochemical staining was performed, and the results revealed increased expression of the pyroptotic factor GSDMD in MGO-induced mice. Treatment with SFN prevented the increase in GSDMD induced by MGO in the aorta (Fig. 9C, D). To determine the underlying mechanisms, we measured the expression of Nrf2 and NLRP3 inflammasome signaling molecules by immunohistochemical staining. The expression of Nrf2 was decreased in MGO-induced mice but was significantly increased by SFN treatment (Fig. 9E, F). Moreover, staining for NLRP3, caspase-1, and IL-1β also revealed that the levels of these proteins were increased by MGO treatment (Fig. 9G–L). NLRP3 inflammasome signaling was significantly decreased by SFN administration (Fig. 9G–L). These results indicate that SFN reverses the MGO-induced downregulation of Nrf2 and upregulation of NLRP3 inflammasome signaling.

## Discussion

MGO accumulation has been reported to induce excessive ROS production, leading to oxidative stress [53, 54], inflammation [55] and cellular death [56], which play prominent roles in the pathogenesis of diabetes and vascular complications [14]. Our previous studies and others have shown that MGO markedly exacerbates oxidative, inflammatory damage and death in HUVECs [16, 19]. SFN is a phytocompound that has antioxidant and anti-inflammatory properties [57], and previous research has demonstrated that SFN can inhibit pyroptosis [58]. However, the specific effect of SFN on MGO-induced pyroptosis in HUVECs and the underlying signaling pathways have remained unclear. Our results confirm that SFN can prevent MGO-induced HUVEC pyroptosis by decreasing oxidative stress, mitochondrial damage, and inflammatory reactions and increasing antioxidant and anti-inflammatory factor levels, which are associated with inhibition of the NLRP3 inflammasome signaling pathway via the activation of the Nrf2/HO-1 signaling pathway.

**Fig. 10:**
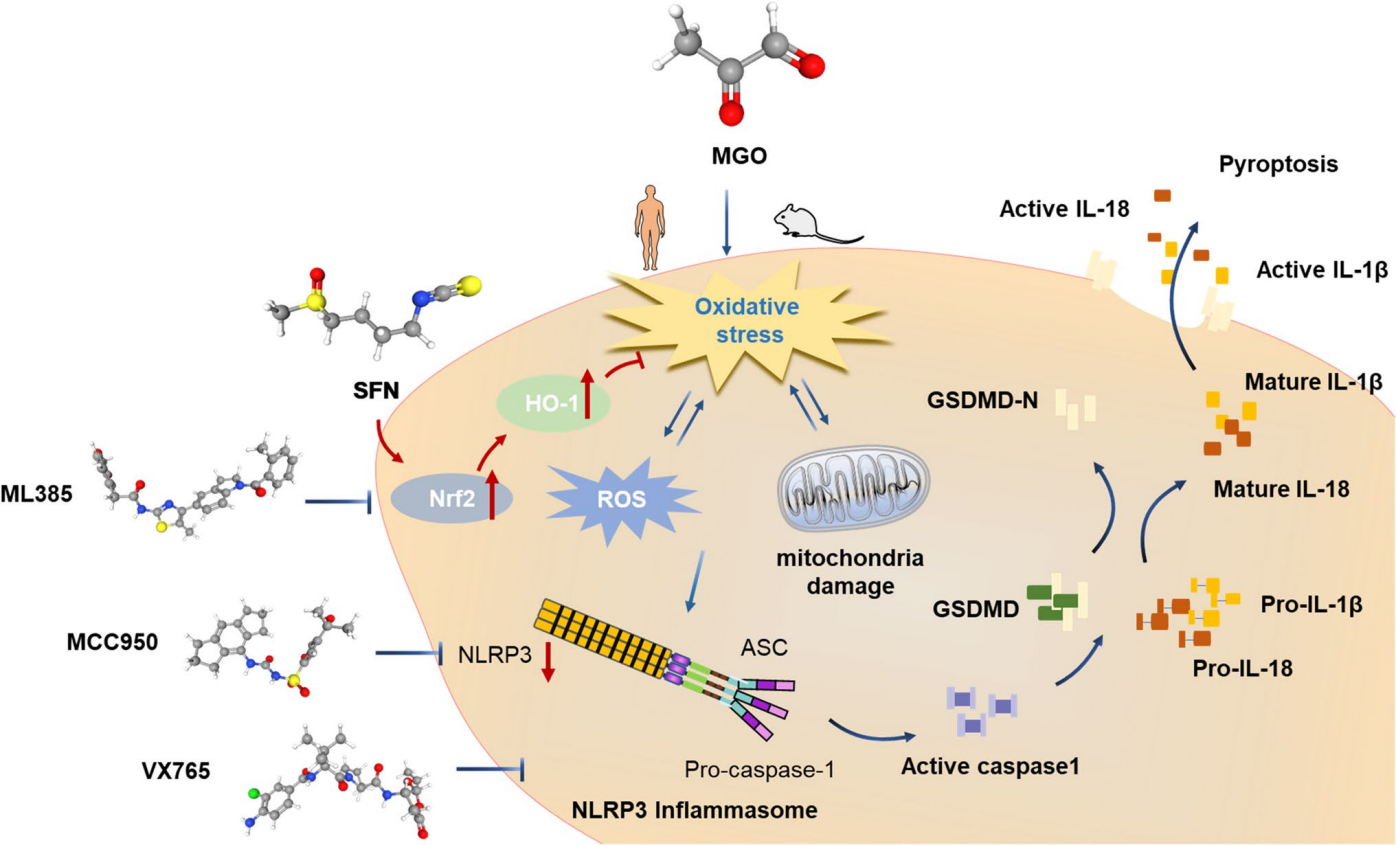
Schematic diagram showing cytoprotective signaling associated with SFN during MGO-induced EC pyroptosis. The molecular mechanisms by which SFN affects MGO-induced dysfunction in human ECs were explored. SFN inhibited the pyroptotic signaling cascades initiated by MGO-induced ROS generation by modulating the Nrf2/HO-1 and NLRP3 inflammasome signaling pathways. Furthermore, SFN effectively protected against MGO-induced oxidative stress, mitochondrial dysfunction, pyroptosis, and inflammation in vitro and in vivo

MGO has been reported to decrease cell viability and induce programmed cell death in several cell types [54, 59, 60], including HUVECs [14, 61]. Our present data clearly demonstrated that MGO significantly reduced HUVEC viability and increased HUVEC pyroptosis and that SFN protected HUVECs from MGO-induced pyroptosis in a dose-dependent manner. Consistent with these observations, pretreatment with SFN prevented MGO-induced HUVEC pyroptosis, inhibited GSDMD expression, and attenuated GSDMD-N cleavage in a dose-dependent manner, thus confirming the protective effects of SFN against MGO-induced pyroptosis. The in vivo effect of SFN on MGO-induced pyroptosis was determined in an MGO-induced vascular injury mouse model, and our data demonstrated that SFN prevented pyroptosis induced by MGO by decreasing GSDMD levels. These findings indicated that SFN effectively prevented HUVEC pyroptosis, providing novel insights into the mechanisms of SFN (Fig. 10).

MGO-induced oxidative stress has significant adverse effects on the antioxidant defense system [62]. The present study revealed that pretreatment with SFN markedly inhibited MGO-induced ROS generation and decreased in SOD, CAT, and GSH-Px activities in HUVECs. Our in vivo data also indicated that SOD, CAT, and GSH-Px levels were decreased by MGO but were only partially restored by pretreatment with SFN. Previous studies have shown that ROS production can activate pyroptosis [8, 49, 63]. Preincubation with the antioxidant NAC notably inhibited ROS generation and MGO-induced pyroptosis. Collectively, these data indicated that SFN inhibits MGO-induced pyroptosis by blocking ROS formation in vivo and in vitro.

Disruption of the MMP, ROS generation [50], and damaged mitochondria [64] are characteristic features of pyroptosis. The present study showed that SFN inhibited MGO-induced depolarization of the MMP and protected against mitochondrial morphological alterations in a dose-dependent manner. The PTP is a large conductance channel in the inner mitochondrial membrane that is activated by intracellular ROS [65]. Our results are consistent with those of previous studies showing that the antioxidative and protective effect of SFN is associated with the inhibition of mitochondrial PTP opening. Moreover, we used an inhibitor of PTP opening (CsA), and our results indicated the that loss of MMP inhibited MGO-induced pyroptosis. MDA is commonly used as a biomarker of oxidative stress [66]. Here, in vitro and in vivo evidence suggest that SFN protects against cell damage by inhibiting the release of MDA. These findings strongly indicated that SFN inhibited MGO-induced pyroptotic biochemical changes in vivo and in vitro by blocking ROS formation and protecting mitochondrial function.

Increased levels of ROS activate Nrf2 signaling, inducing the expression of antioxidant enzymes such as HO-1, which protects cells against oxidative stress [67, 68]. In the present study, pretreatment with SFN further increased Nrf2/HO-1 levels in a dose-dependent manner, thus suppressing MGO-induced pyroptosis in vitro and in vivo. Moreover, our results demonstrated that the Nrf2 inhibitor ML385 notably abolished the protective effect of SFN on MGO-induced HUVEC pyroptosis. In addition, the NLRP3 inflammasome, which is the best-characterized inflammasome, can be activated by a broad range of stimuli, including mitochondrial dysfunction and ROS generation [69, 70]. Our present study showed that an increase in Nrf2 (ML385) alleviated oxidative stress, and NLRP3 inflammasome activation was decreased in HUVECs. Moreover, we used an inhibitor of NLRP3 (MCC950), and our results indicated that SFN suppressed pyroptotic cell death in an NLRP3-dependent manner, since pretreatment with MCC950 could enhance the SFN-induced reduction in MGO-induced pyroptosis in HUVECs. In addition, by using an inhibitor of caspase-1(VX765), our experimental results indicated that inhibition of caspase-1 inhibited MGO-induced pyroptosis. Taken together, our findings demonstrated that SFN could protect against pyroptosis by inhibiting the NLRP3 inflammasome (NLRP3, ASC, and caspase-1) signaling pathway by activating Nrf2/HO-1 signaling.

Our previous studies have confirmed that MGO also induces apoptosis of ECs via increased oxidative stress and inflammation. It would be interesting to disect out if after MGO treatment in ECs the inclination is more towards selecting an apoptotic pathway or pyroptotic pathway to recognize the pathophysiological relevance of the study. Although MGO is known to induce various forms of cell death, it may exhibit some propensity. Cepas V et al. [71] showed that MGO greatly reduces cell viability, leading to caspase-mediated apoptosis that affects approximately 85.35% of total cells. In addition, Ishida E et al. [72] found that MGO induces cell death by generating ROS in vascular ECs, which is inhibited by ASC deficiency. Meanwhile, treatment with MGO resulted in a significant upregulation of inflammasome-associated proteins, indicating that NLRP3 inflammasomes are integral to MGO-induced cell death. Combined with our previous findings, it can be hypothesized that MGO induces oxidative stress in ECs, leading to apoptosis, while at the same time it can cause an inflammatory response that induces cellular pyroptosis. These results suggest that these two modes of death coexist, but in different proportions and with a tendency. Notably, Zhou S et al. [73] found that reduction of MGO induced the conversion of apoptosis to pyroptosis and carried out pyroptosis-induced immunotherapy based on pyroptosis to significantly eliminate tumors. The conversion of apoptosis to pyroptosis due to reduced MGO content suggests that it may be possible to regulate the type of cell death by adjusting the concentration of MGO. In our study protocol, the MGO concentration regimen was aligned with the results of this study, but the specific regulatory mechanisms require further investigation.

In conclusion, our study is the first to demonstrate that SFN effectively protects against MGO-induced oxidative stress, mitochondrial dysfunction, inflammation, and pyroptosis in vitro and in vivo. Specifically, SFN prevents the pyroptotic signaling cascades initiated by MGO-induced ROS and inflammatory cytokines by modulating the Nrf2/HO-1 and NLRP3 inflammasome signaling pathways (Fig. 9). This compelling evidence expands our understanding of the benefits and pharmacological applications of SFN and provides novel insights for the development of strategies to preserve endothelial function in vascular diseases.

## Supplementary Information

Below is the link to the electronic supplementary material.

Supplementary file1 (TIFF 64 KB)

## Data Availability

The datasets used and/or analyzed during the current study are available from the corresponding author on reasonable request.

## Abbreviations

MGO: Methylglyoxal
ECs: Endothelial cells
NLRP3: The NOD-like receptor 3
LDH: Lactate dehydrogenase
Nrf2: Nuclear transcription factor erythroid 2-related factor 2
HO-1: Heme oxygenase
SFN: Sulforaphane
ROS: Reactive oxygen species
MMP: Mitochondrial membrane potential
DM: Diabetes mellitus
AGE: Advanced glycation end-product
GSDMD: Gasdermin-D
GSDMD-N: N-terminal cleaving product
PTPs: Permeability transition pores
